# Insecticide resistance mechanisms associated with different environments in the malaria vector *Anopheles gambiae*: a case study in Tanzania

**DOI:** 10.1186/1475-2875-13-28

**Published:** 2014-01-25

**Authors:** Theresia E Nkya, Idir Akhouayri, Rodolphe Poupardin, Bernard Batengana, Franklin Mosha, Stephen Magesa, William Kisinza, Jean-Philippe David

**Affiliations:** 1 Laboratoire d'Ecologie Alpine, UMR CNRS-Université de Grenoble 5553, BP 53, 38041, Grenoble cedex 09, France; 2 National Institute of Medical Research of Tanzania, Amani Medical Research Centre, P. O. Box 81, Tanga, Muheza, Tanzania; 3 Liverpool School of Tropical Medicine, Vector Group. Pembroke place, Liverpool L35QA, UK; 4 KCM College of Tumaini University, P. O. Box. 2240, Moshi, Tanzania; 5 RTI International-Tanzania, P.O.Box 369, Dar es Salaam, Tanzania

**Keywords:** Malaria vector, Mosquitoes, Anopheles, Insecticide resistance, Agriculture, Pollution, Detoxification enzymes, Kdr mutation, Environment

## Abstract

**Background:**

Resistance of mosquitoes to insecticides is a growing concern in Africa. Since only a few insecticides are used for public health and limited development of new molecules is expected in the next decade, maintaining the efficacy of control programmes mostly relies on resistance management strategies. Developing such strategies requires a deep understanding of factors influencing resistance together with characterizing the mechanisms involved. Among factors likely to influence insecticide resistance in mosquitoes, agriculture and urbanization have been implicated but rarely studied in detail. The present study aimed at comparing insecticide resistance levels and associated mechanisms across multiple *Anopheles gambiae sensu lato* populations from different environments.

**Methods:**

Nine populations were sampled in three areas of Tanzania showing contrasting agriculture activity, urbanization and usage of insecticides for vector control. Insecticide resistance levels were measured in larvae and adults through bioassays with deltamethrin, DDT and bendiocarb. The distribution of *An. gambiae* sub-species and pyrethroid target-site mutations (*kdr*) were investigated using molecular assays. A microarray approach was used for identifying transcription level variations associated to different environments and insecticide resistance.

**Results:**

Elevated resistance levels to deltamethrin and DDT were identified in agriculture and urban areas as compared to the susceptible strain Kisumu. A significant correlation was found between adult deltamethrin resistance and agriculture activity. The subspecies *Anopheles arabiensis* was predominant with only few *An. gambiae sensu stricto* identified in the urban area of Dar es Salaam. The L1014S *kdr* mutation was detected at elevated frequency in *An gambiae s.s*. in the urban area but remains sporadic in *An. arabiensis* specimens. Microarrays identified 416 transcripts differentially expressed in any area versus the susceptible reference strain and supported the impact of agriculture on resistance mechanisms with multiple genes encoding pesticide targets, detoxification enzymes and proteins linked to neurotransmitter activity affected. In contrast, resistance mechanisms found in the urban area appeared more specific and more related to the use of insecticides for vector control.

**Conclusions:**

Overall, this study confirmed the role of the environment in shaping insecticide resistance in mosquitoes with a major impact of agriculture activities. Results are discussed in relation to resistance mechanisms and the optimization of resistance management strategies.

## Background

Recent years have shown a decline in malaria prevalence attributed to efficient vector control strategies implemented in endemic areas [1]. In Tanzania, malaria is still a major public health problem, leading to high mortality and morbidity [2] and claiming more than one third of all deaths in children under five years old [3]. Members of the *Anopheles gambiae* complex are the main malaria vectors in Tanzania, with *An. gambiae sensu stricto* (referred to as *An. gambiae s.s*.) being endophagic and *Anopheles gambiae arabiensis* (referred to as *An. arabiensis*) being more exophagic [4]. *Anopheles funestus* is also present in southern Tanzania mainly during dry seasons [5].

As in other African countries, control programmes are based on the application of chemical insecticides by the use of insecticide-treated nets (ITNs) or indoor residual spraying (IRS). Current programmes are largely dependent on synthetic pyrethroids, which are the only WHO-recommended insecticides for ITNs [6]. DDT and bendiocarb are also used for IRS in several African countries, including Tanzania [7-9]. Both DDT and pyrethroids target the voltage-gated sodium channel (VGSC) in the mosquito central nervous system [10], while carbamates block the degradation of the neuromediator acetylcholine by inhibiting the acetylcholinesterase [11]. Following years of intensive usage, insecticide efficacy is now threatened by the rise of resistance in target populations. Such a phenomenon is occurring throughout the whole African continent and spreads at a rapid rate [12-15]. In Tanzania, reports of insecticide resistance in malaria vectors were rare but the situation is now changing with the rise of pyrethroid and DDT resistance in several regions [9,16,17]. Indeed, in 2011, a national wide survey indicated that most *An. gambiae* populations were still susceptible to carbamates and organophosphates while resistance to DDT and pyrethroids was rising in some areas [16].

In malaria vectors, resistance can be the consequence of mutations in the target proteins (target-site insensitivity), a lower penetration or sequestration of the insecticide, or an increased biodegradation of the insecticide due to enhanced detoxification activities (metabolic resistance). Resistance to pyrethroids appears to rely mainly on the ‘knock down resistance’ target-site mutations (*kdr*) and metabolic resistance mechanisms, although other mechanisms, such as cuticle alteration have also been evoked [18-20]. *Kdr* mutations and elevated levels of detoxification enzymes also confer resistance to DDT while carbamate resistance can be conferred by *ace1* mutation and detoxification [21-23].

The recurrent use of insecticides for vector control is believed to be the main cause of resistance in mosquito populations [15,24-26]. Concomitantly, the use of insecticides for the control of crop pests in agriculture and the presence of pollutants in urban and industrial areas have been suggested to play a significant role in selecting for insecticide resistance in mosquitoes [18]. Indeed, most pesticides used in agriculture have the same targets as those used for vector control, and can therefore select for resistance mechanisms in mosquitoes breeding in areas of intense agriculture activities [27-30]. Such cross-selections may also occur in urban environments where rapid urbanization promotes small-scale vegetable farming as source of livelihood and income and has led to an uncontrolled use of pesticides [31,32]. In addition, several studies showed that urban pollutants can affect mosquito detoxification system leading to an enhanced tolerance to insecticides [33-38]. In Tanzania, urbanization is spreading dramatically around large cities, and several areas show an intense agriculture activity [39] potentially affecting the resistance status of malaria vectors toward insecticides. Populations of *An. gambiae* from an area of intense agriculture in lower Moshi (Kilimanjaro region) were found highly resistant to pyrethroids [17]. As in other African countries, elevated insecticide resistance levels of malaria vectors have also been identified in Tanzanian urban areas [16,27,32,35].

In this context, the present study aimed at investigating insecticide resistance levels and associated mechanisms in malaria vectors from different environments in East Tanzania. To achieve this, nine *Anopheles* populations were sampled across three large areas characterized by different environments: *i*) an agriculture area that has a long history of pesticide use in coffee and rice farms and low ITN coverage, *ii*) an urban area from a large city with high anthropogenic pollutants and use of insecticides for vector control and *iii*) a low pollution area characterized by a limited use of pesticides for agriculture and vector control and a low urbanization. Resistance levels of each population at both larval and adult stages were determined by bioassays with deltamethrin, DDT and bendiocarb. The frequency of *An. gambiae s.s*. and *An. arabiensis* were assessed together with the presence of *kdr* mutations using PCR assays. A genome-wide microarray approach was used to compare transcriptome variations between each area and identify genes whose expression profiles were linked to insecticide resistance. These results are discussed in relation to known resistance mechanisms and their relationship with different environments.

## Methods

### Study sites and mosquito collection

**Figure 1:**
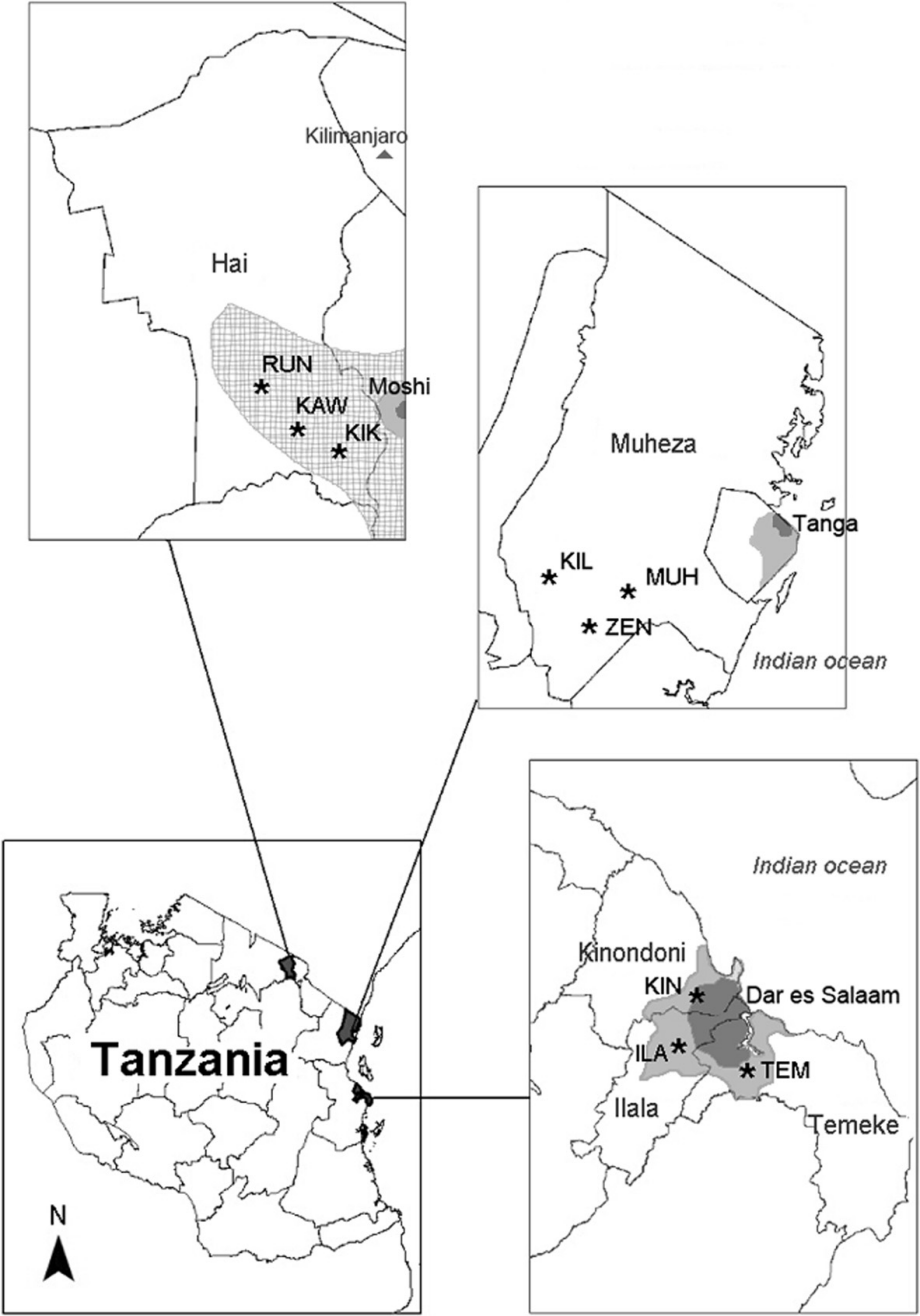
**Geographical location of sampled populations.** Populations are indicated by stars. Urbanization level is indicated by light (medium) and dark (high) grey. Intense agriculture area is shown by cross-hatching. District and city names are indicated.

This study took place in three large geographical areas in the east of Tanzania (Figure 1). These three areas were characterized by different environments as follows: i) urban area ii) agriculture area and iii) low pesticide area depending on urbanization, agriculture and vector control activities. Three populations separated by distances from 10 to 25 Km were studied in each area. Each population consisted of several breeding sites.

Urban area was characterized by a high degree of human activity, the presence of urban and industrial pollutants and a high ITN coverage. Dar es Salaam was chosen as the urban area because of its density (population ~ 2.5 million) and its intense economic activity. Three populations were sampled in different city areas; Ilala (ILA), Temeke (TEM) and Kinondoni (KIN). Agriculture area was characterized by an intense agriculture activity with a recurrent use of pesticides on crops and a low ITN coverage. The lower Hai district, below Kilimanjaro Mount and near Moshi was chosen because of its low population density and intense agriculture activity requiring the use of pesticides (rice fields, sugar cane and coffee). Three populations were sampled across the area; Rundugai (RUN), Kikafu (KIK) and Kawaya (KAW). Low-pesticide area was characterized by low urbanization, small-scale farming with limited use of pesticides and a medium to high ITN coverage. The Muheza district, in the Tanga region was chosen as low pesticide area. Three populations were sampled across the area; Zeneti (ZEN), Kilulu (KIL) and Muheza estate (MUH).

Various ecological parameters were estimated from each population (Additional file 1) including breeding site type, surrounding area type (5 km range), surrounding area ITN coverage (5 km range data based on community interview), use of pesticide in agriculture (based on interview of farmers and pesticide shop owners), urbanization, malaria prevalence [40] and vegetation coverage. The five last parameters were quantified as follows for pairwise correlation studies (Additional file 2): absent (1), very low (2), low (3), medium (4), high (5).

Mosquitoes were collected from each population in 2011 during the long and short rain seasons. *Anopheles* F_0_ larvae were collected and brought back to the laboratory for rearing in standardized conditions (natural photoperiod, 30°C, larvae feed on Tetramin® fish food). F_0_ larvae were reared to adulthood, allowed to mate and females were blood fed on rabbits to obtain F_1_ eggs. In order to avoid transient effects due to environmental variations, only F_1_ larvae and F_1_ adults reared in standardized conditions were used for bioassays and molecular analyses. Mosquitoes used for molecular analyses were stored individually in RNAlater (Ambion) at -20°C.

### Bioassays with insecticides

Larvae and adult bioassays were performed following WHO protocols. Larvae bioassays were carried out using the pyrethroid deltamethrin, the organochlorine DDT and the carbamate bendiocarb and third stage larvae of each population. Four replicates per concentration and 6 concentrations in the activity range of each insecticide were used (N = 50 per replicate per concentration). Larval mortality was recorded after 24 h exposure to each insecticide and LC_50_ (lethal concentration required for killing 50% of larvae) calculated using a probit approach.

Adult bioassays were performed on 3-5 days-old females following WHO guidelines with plastic test tubes and insecticide impregnated papers at the following concentrations: 4% DDT, 0.05% deltamethrin, and 0.1% bendiocarb. Four replicates of 10-25 mosquitoes were exposed to each insecticide for 60 minutes. For deltamethrin and DDT, the number of knockdown (KD) mosquitoes was recorded after 5, 10, 20, 30, 45 and 60 minutes exposure and KDT_50_ (time required for knocking down 50% of individuals) estimated. After 1 h exposure mosquitoes were provided with 10% glucose solution and allowed recovering for 24 h before recording mortality. For bendiocarb, mosquitoes were exposed for varying durations (5, 10, 20, 30, 45 or 60 min). For each exposure time, adults were allowed to recover as described above before measuring mortality. Controls consisted of larvae or adults from each population not exposed to any insecticide. Mortality was corrected using Abbot Formula when the mortality rate of controls was between 5-20%. Two fully susceptible reference strains were also tested in parallel: the Kisumu strain (*An. gambiae s.s*. S form isolated from west Kenya) and the Ifakara strain (*An. arabiensis* isolated from central Tanzania). For each insecticide, larval resistance ratios (RR_50_) consisted of LC_50_ obtained for each population divided by LC_50_ obtained with the Kisumu strain. For adults, RR_50_ consisted of KDT_50_ of each population divided by the KDT_50_ of the Kisumu strain. As bendiocarb KDT_50s_ could not be estimated because of high mortality, % mortality after 10 min and 24 h recovery was used for comparing populations.

### Correlation between resistance levels and ecological factors

Correlation between environmental data (ITN coverage, agriculture intensity, urbanization intensity, malaria prevalence and vegetation coverage), insecticide resistance levels (larval LC_50_, larval LC_95_, adult KDT_50_, adult KDT_95_ and adult % mortality to each insecticide), *kdr* mutation frequency and the frequency of *An. gambiae s.s*. and *An. arabiensis* specimens were investigated across the nine field populations. Pearson’s correlation coefficients were calculated for all possible pairwise comparisons and then subjected to a *t*-test using the formula t = r × √(n-1) / √(1-r^2^). P values were then adjusted using Holm’s multiple testing correction. Correlations showing a r ≥ 0.8 or ≤ -0.8 and an adjusted P value ≤ 0.05 were considered significant.

### Species identification and kdr genotyping

For each population, genomic DNA samples (gDNA) from 50 larvae and 50 adults were extracted individually. gDNA was extracted by grinding and heating the last larval segment (larvae) or mosquito legs (adults) at 65°C for 30 minutes in 100 μl Bender buffer (0.1 NaCl, 0.2 M sucrose, 0.1 M tris-HCL pH 7.5, 0.05 M EDTA pH 9.1, 0.5% SDS) according to the method described by Collins *et al*. [41]. These individual gDNA samples was used for discriminating between *An. gambiae s.s*. and *An. arabiensis* following the PCR-based method described by Scott *et al*. [42]. The same samples were used for estimating the frequency of the *L1014F* and *L1014S kdr* alleles using the TaqMan® PCR diagnostic assays described in Bass *et al*. [43] and a MX3005P qPCR system (Agilent).

### Gene expression study with microarrays

Because *An. arabiensis* represented the majority of individuals and in order to avoid potential bias due to different species ratios across populations, only individuals identified as *An. arabiensis* were used for RNA extraction and subsequent gene expression analyses. Total RNA was extracted from pools of 25 three days-old adult females from each population and three replicates of 25 individuals from the Ifakara susceptible strain using the RNAqueous-4PCR kit (Ambion). Total RNAs were treated with DNaseI (Ambion) to remove any genomic DNA contaminant. The quantity and integrity of RNA was analysed using a NanoDrop ND1000 Spectrophotometer (NanoDrop Technologies) and a 2100 Bioanalyzer (Agilent). Hundred ng total RNA were used for cDNA synthesis and T7-RNA amplification with Cy3-CTP or Cy5-CTP labelling using the ‘Two-colors Low Input Quick Amp’ labelling kit (Agilent Technologies) following manufacturer’s instructions. Labelled cRNA were purified and their Cy3/Cy5 specific activity and quantity measured using a Nanodrop ND1000 (NanoDrop Technologies) and a 2100 Bioanalyzer (Agilent). Cy3- or Cy5-labelled cRNAs obtained from 3 biological replicates of the susceptible Ifakara strain were pooled together in equal quantity to build a common reference for all hybridizations. cRNAs from each population were hybridized versus cRNA of the reference (300 ng/sample) using the microarray AGAM 15 K (Agilent) representing more than 15 K *An. gambiae* transcripts as described in Mitchell [44]. After hybridization, non-specific probes were washed off according to manufacturer's instructions and slides were scanned with an Agilent G2205B microarray scanner. Spot finding, signal quantification and Lowess normalization were performed automatically using the Agilent Feature Extraction software (Agilent). Two hybridizations where Cy3 and Cy5 dyes were swapped were performed for each population for a total of 18 hybridizations. Normalized data were loaded into Genespring GX version 12.5 (Agilent). Spots showing a signal-to-noise ratio > 2 for both colours were flagged as detected and only probes detected in 50% of hybridizations were considered for further analysis.

Gene expression analysis was first performed at the area level (3 populations per area: ‘Urban’, ‘Agriculture’ and ‘Low pesticide’). For each area, mean expression ratios compared to the reference strain Ifakara were calculated, log transformed and submitted to a one sample student’s *t*-test against 0 (equal transcription compared to reference strain) followed by Benjamini and Hochberg multiple testing correction. Transcripts showing a mean transcription ratio > 3 fold in either direction and a corrected P value < 0.001 were considered significantly differentially transcribed. These 416 transcripts were then submitted to hierarchical clustering analysis based on their expression profile across all areas. Clustering was based on Euclidean distance between log_2_ ratios versus reference in each area and complete linkage algorithm. Transcripts belonging to each main cluster were then submitted to a Gene Ontology (GO) term enrichment analysis using Bingo version 2.4 [45]. Enrichment was assessed by a hypergeometric test followed by Benjamini and Hochberg multiple testing correction and restricted to the GO category ‘Molecular function’. In each cluster, GO terms showing a corrected P value < 0.05 were considered significantly enriched compared to those represented in all detected transcripts. Significantly enriched GO terms were then graphically represented as networks using Cytoscape version 2.8.2 (cytoscape.org).

The 27 differentially expressed transcripts encoding genes potentially involved in insecticide resistance (insecticide targets, cuticle proteins, detoxification enzymes, ABC transporters, red/ox enzyme…) were then submitted to a focused clustering analysis as described above.

As differential tolerance to deltamethrin was observed between the nine studied populations, correlations between the expression profile of particular transcripts and deltamethrin RR_50_ or *kdr* mutation frequency were investigated within all detected genes across all populations. Transcripts showing transcription profile with a correlation coefficient > 0.85 or < -0.85 to deltamethrin RR_50_ or *kdr* mutation frequency were retained and submitted to a focused clustering analysis as described above.

### RT-qPCR validation

Reverse-transcription quantitative PCR (RT-qPCR) was used to confirm the expression profile of the cytochrome P450 monooxygenase CYP6P3 (AGAP002865) and the cuticle protein AGAP000987 across populations. Specific primers targeting each transcript were designed using Beacon Designer 7.02 and their specificity checked using vectorbase Blast function. Total RNA samples used for RT-qPCR were identical to those used for microarrays. Experimental procedures used for reverse transcription and real-time PCR are described in Poupardin [46]. Data analysis was performed according to the ΔΔ^Ct^ method taking into account PCR efficiency [47] and using the housekeeping genes encoding L8 (AGAP005802) and S7 (AGAP010592) ribosomal proteins. Four replicates were performed per population and results were expressed as mean transcription ratio relative to the reference strain Ifakara.

## Results

### Mosquito species distribution

A total of 547 *Anopheles* larvae and adults from the nine sampled populations were used for species identification by PCR [40]. Most individuals belong to *An. gambiae* complex with only a few samples identified as other *Anopheles* species in various populations (Table 1). *An. arabiensis* was predominant in most populations and *An. gambiae s.s*. was mostly found in the urban area of Dar Es Salaam representing up to 50% in the ILA population.

**Table 1:** Mosquito species distribution

**Area**	**Pop**	**Larvae**				**Adults**				**Mean**	
		**N**	**Frequency (%)**			**N**	**Frequency (%)**			**Frequency (%)**	
			***An. gamb***	***An. arab***	**Other**		***An. gamb***	***An. arab***	**Other**	***An. gamb***	***An. arab***
Lab strains	KIS	50	100	0	0	50	100	0	0	100	0
	IFA	50	0	100	0	50	0	100	0	0	100
Urban	ILA	50	6.0	70	24	50	50	30	20	28	50
	TEM	50	0	100	0	45	0	93.3	6.7	0	96.7
	KIN	50	0	89.5	10	47	17	32	6.3	8.5	60.8
Non-polluted	ZEN	50	0	94	6	50	0	98	2	0	96
	KIL	49	6.1	71.4	22.4	49	0	100	0	3.5	85.7
	MUH	50	0	96	4	50	0	100	0	0	98
Agriculture	RUN	50	0	100	0	50	0	98	2	0	99
	KIF	50	0	100	0	50	0	100	0	0	100
	KAW	50	0	98	2	49	0	92	8	0	95

*An. gamb*: *Anopheles gambiae senus stricto*.

*An. Arab*: *Anopheles arabiensis*.

### Insecticide resistance levels

Larval bioassays revealed low resistance levels to deltamethrin, DDT and bendiocarb compared to the susceptible Kisumu strain (Table 2). Deltamethrin resistance ranged from 3.8 to 15.4 fold. One could note that a resistance ratio (RR_50_) of 3.8 was observed for the susceptible Ifakara strain suggesting that populations showing a RR_50_ below 4 fold can be considered as susceptible. Populations from the urban and agricultural areas showed elevated resistance to deltamethrin with a mean RR_50_ of 10.9 fold and 8.2 fold respectively. Regarding DDT, resistance was slightly elevated in the urban area (mean RR_50_ 3.1 fold) but remain low in other areas. Overall, bendiocarb resistance was low although the KIK population from the agriculture area showed a 3.5 fold increased resistance.

**Table 2:** **Resistance of larvae to deltamethrin**, **DDT and bendiocarb**

**Area**	**Pop**	**Deltamethrin**		**DDT**		**Bendiocarb**	
		**LC**_**50**_ (**95**% **CI**)	**RR**_**50**_ (**95**% **CI**)	**LC**_**50**_ (**95**% **CI**)	**RR**_**50**_ (**95**% **CI**)	**LC**_**50**_ (**95**% **CI**)	**RR**_**50**_ (**95**% **CI**)
Lab strains	KIS	6.8 (2.6-10.5)	1	407.6 (284.7-786.8)	1	29.1 (20.5-41.6)	1
	IFA	22.9 (21.2-24.8)	3.8 (2.3-8.1)	135.2 (115.9-155.1)	0.3 (0.2-0.4)	29.6 (23.7-36.5)	1.0 (0.9-1.2)
Urban	ILA	49.3 (39.5-60.3)	7.3 (5.7-15.1)	738.0 (582.2-1011.0)	**1.8** (1.3-2.0)	73.5 (50.4-132.0)	**2.5** (2.5-3.2)
	TEM	104.6 (70.0-213.0)	**15.4** (20.2-26.7)	832.5 (654.1-1154.0)	**2.0** (1.5-2.3)	42.9 (31.8-61.5)	**1.5** (1.5-1.5)
	KIN	67.7 (50.4-101.3)	**10.0** (9.6-19.2)	722.8 (594.0-922.7)	**1.8** (2.1-1.2)	82.5 (60.1-137.2)	**2.8** (2.9-3.3)
Non-polluted	ZEN	27.0 (17.6-36.6)	4.0 (3.4-6.7)	655.9 (518.9-886.3)	1.6 (1.0-1.8)	43.6 (27.4-83.9)	**1.5** (1.3-2.0)
	KIL	27.0 (16.9-38.8)	4.0 (3.7-6.4)	773.5 (651.7-930.6)	**1.9** (1.2-2.9)	58.9 (39.0-111.4)	**2.0** (1.9-2.7)
	MUH	30.4 (21.6-39.5)	4.5 (3.7-8.2)	633.8 (503.6-847.4)	1.6 (1.0-1.8)	63.0 (41.0-125.6)	**2.2** (2.0-3.0)
Agriculture	RUN	34.6 (26.9-45.0)	5.1 (4.3-10.3)	175.6 (147.9-203.4)	0.4 (0.3-0.5)	37.5 (29.8-47.1)	1.3 (1.1-1.5)
	KIK	103.4 (83.5-136.8)	**15.2** (13.0-31.8)	172.0 (132.1-213.2)	0.4 (0.3-0.5)	103.4 (83.5-136.8)	**3.5** (3.3-4.1)
	KAW	30.0 (18.2-49.8)	4.4 (4.3-6.9)	164.6 (132.3-197.6)	0.4 (0.2-0.5)	34.8 (26.9-45.6)	1.2 (1.1-1.3)

LC_50_: lethal concentration for 50% of individuals (μg/L). RR_50_: resistance ratio based on LC50 values. RR_50_ values with CI_95_ intervals not overlapping those of both susceptible strains are shown in bold. Bioassays were performed on *Anopheles gambiae sensu lato* larvae.

Adult bioassays confirmed the moderate resistance levels of populations from urban and agriculture areas to deltamethrin with mean RR_50_ of 3.1 fold and 5.6 fold respectively and a mortality rate after 1 h exposure to WHO diagnostic dose between 84 and 100% (Table 3). As for larvae, DDT resistance was slightly elevated in urban area (2.2 fold in TEM). All tested populations showed 100% mortality following 1 h exposure to bendiocarb WHO diagnostic dose. However, when considering shorter exposure times, most field populations showed a better survival than the two susceptible populations suggesting a slight increased resistance to this insecticide (Table 3).

**Table 3:** **Resistance of adults to deltamethrin**, **DDT and bendiocarb**

**Area**	**Pop**	**Deltamethrin**			**DDT**			**Bendiocarb**
		**KDT**_**50**_ (**95**% **CI**)	**RR**_**50**_ (**95**% **CI**)	% **Mort** (**SE**)	**KDT**_**50**_ (**95**% **CI**)	**RR**_**50**_ (**95**% **CI**)	% **Mort** (**SE**)	% **Mort** (**SE**)
Lab strain	KIS	5.7 (5.0-6.2)	1.0	100 (0.0)	24.2 (20.8-27.8)	1.0	100 (0.0)	95 (2.3)
	IFA	12.4 (11.4-13.5)	2.2 (2.1-2.3)	100 (0.0)	15.7 (6.7-24.9)	0.6 (0.3-0.9)	100 (0.0)	95 (1.7)
Urban	ILA	19.7 (17.6-21.7)	**3.5** (3.5-3.5)	100 (0.0)	34.7 (32.6-36.8)	**1.4** (1.3-1.6)	94.5 (2.2)	**33** (1.4)
	TEM	22.5 (19.6-25.8)	**4.0** (3.9-4.1)	98 (1.2)	52.3 (26.0-32.2	**2.2** (1.2-1.2)	98 (1.2)	54 (1.2)
	KIN	10.0 (8.4-11.6)	1.8 (1.7-1.8)	100 (0.0)	27.8 (25.8-30.1)	**1.2** (1.1-1.1)	95 (3.0)	**20** (4.9)
Non-polluted	ZEN	8.0 (5.8-10.1)	1.4 (1.2-1.6)	100 (0.0)	28.6 (26.7-30.6)	**1.2** (1.1-1.3)	95 (3.2)	**46** (7.0)
	KIL	7.7 (5.8-9.6)	1.4 (1.1-1.5)	100 (0.0)	26.7 (24.7-28.6)	1.1 (1.0-1.2)	96 (2.3)	55 (7.0)
	MUH	8.9 (6.9-10.8)	1.6 (1.4-1.7)	100 (0.0)	26.5 (24.5-28.6)	1.1 (1.0-1.2)	95 (3.5)	63 (6.6)
Agriculture	RUN	32.8 (30.6-34.9)	**5.8** (5.6-6.1)	86 (4.8)	22.1 (19.3-24.7)	0.9 (0.9-0.9)	100 (0.0)	52 (4.3)
	KIK	32.7 (30.8-34.6)	**5.8** (5.5-6.2)	84 (4.6)	22.1 (21.1-23.0)	0.9 (0.8-1.1)	100 (0.0)	54 (7.4)
	KAW	30.2 (28.5-32.0)	**5.3** (5.1-5.7)	87 (5.3)	22.9 (21.3-24.3)	0.9 (0.9-1.0)	100 (0.0)	60 (2.8)

KDT_50_: Knock down time for 50% of individuals (min). RR_50_: resistance ratio based on KDT_50_ values. RR_50_ values with CI_95_ intervals not overlapping those of both susceptible strains are shown in bold. For bendiocarb, % mortality after 10 min exposure is indicated with populations showing less than 50% mortality in bold. Bioassays were performed on *Anopheles gambiae sensu lato* adults.

### *Kdr* mutation frequency

Although both *L1014F* (*kdr west*) and *L1014S* (*kdr east*) mutations were screened, only the *L1014S kdr* ‘*east*’ mutation was detected (Table 4). This *kdr* mutation was present at high frequency in *An. gambiae s.s*. while only one heterozygous *kdr* individual was detected in *An. arabiensis* in the KIL population. In consequence, *kdr* mutation frequency was moderate in urban populations containing *An. gambiae s.s*. (ILA and TEM) and almost absent in populations from other areas.

**Table 4:** L1014S kdr mutation frequencies

**Area**	**Pop**	**Specie**	**Genotype**				
			LL	LS	SS	S frequency (%)	N
Urban	ILA	*An. gambiae s.s*.	3	3	22	84	28
		*An. arabiensis*	45	0	0	0	45
	TEM	*An. gambiae s.s*.	0	0	0	0	0
		*An. arabiensis*	92	0	0	0	92
	KIN	*An. gambiae s.s*.	3	3	5	59	11
		*An. arabiensis*	7	0	0	0	79
Non-polluted	ZEN	*An. arabiensis*	91	0	0	0	91
	KIL	*An. arabiensis*	79	1	0	0.6	79
	MUH	*An. arabiensis*	92	0	0	0	92
Agriculture	RUN	*An. arabiensis*	100	0	0	0	100
	KIK	*An arabiensis*	100	0	0	0	100
	KAW	*An arabiensis*	95	0	0	0	95

LL; fully susceptible individuals, LS; heterozygote resistance individuals,

SS; homozygote resistance individuals.

### Correlation between resistance levels and ecological factors

The only significant correlation found between ecological factors and resistance data involved deltamethrin resistance and agriculture (Additional file 2) where adult mortality after 1 h exposure to deltamethrin was negatively correlated to agriculture intensity (higher tolerance in agriculture area, r = -0.95 adjusted P value = 0.0018). Significant correlations were also found between deltamethrin resistance and DDT resistance (Additional file 2). As expected, a strong positive correlation was found between the frequency of the *L1014S kdr* mutation and the presence of *An. gambiae s.s*.

### Transcription profiling using the *Anopheles gambiae* 15 k microarray

By using the *An. gambiae* 15 K microarray and the susceptible strain Ifakara as reference, the expression profiles of *An. arabiensis* females from each population were compared. This analysis allowed detecting 8280 probes corresponding to 7904 distinct transcripts showing a high and reproducible signal-to-noise ratio across all populations.

**Figure 2:**
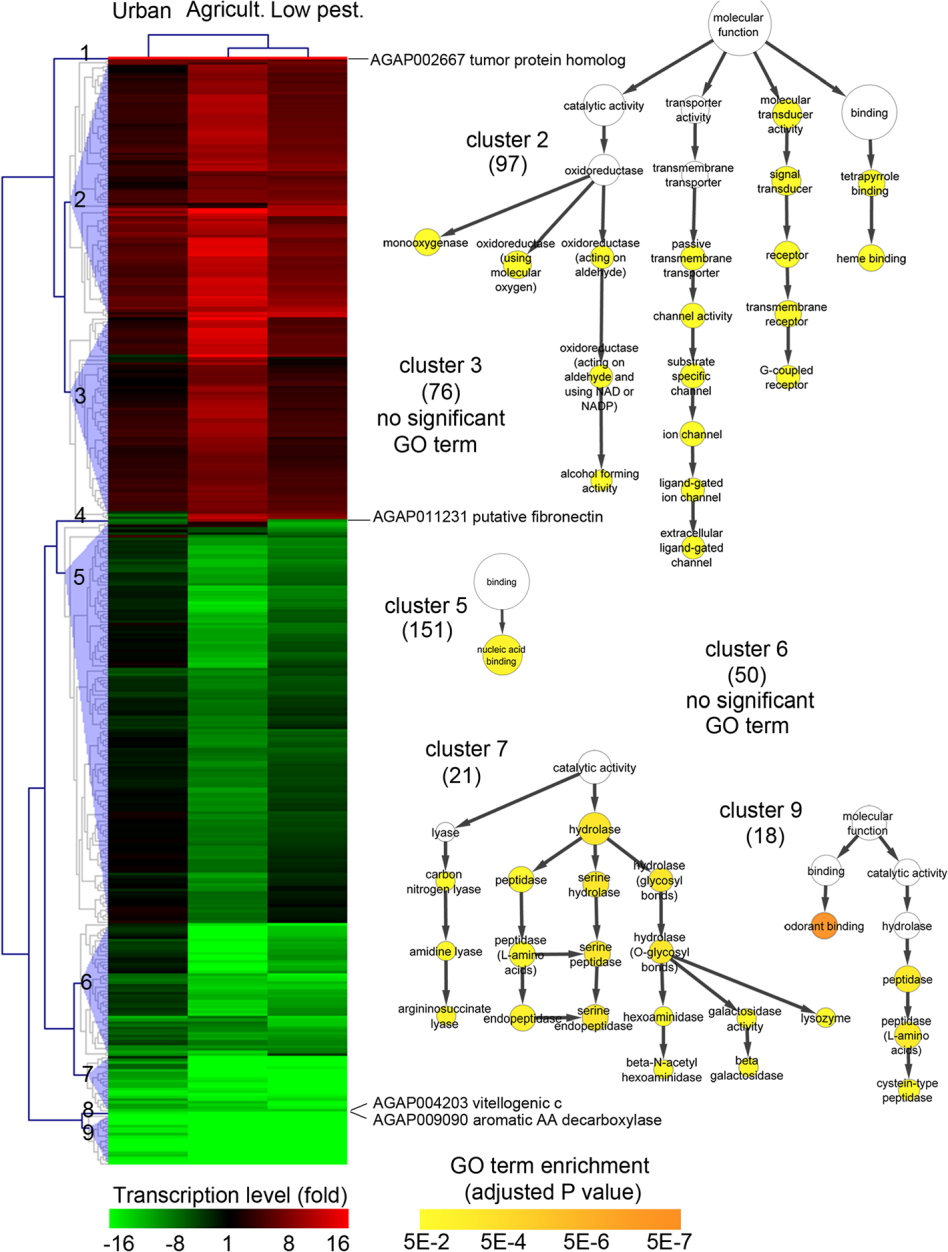
**Genes differentially transcribed in any area.** Clustering analysis was performed on the 416 transcripts showing a significant differential expression in any area compared to the reference strain Ifakara (P ≤ 0.001 and FC ≥ 3). Clustering of transcripts and conditions were based on differences between mean Log_2_ ratios in each area versus reference using Euclidean distance and complete linkage algorithm. Colour scale (green to red) indicates fold change versus reference strain. The number of transcripts represented in each cluster is shown within bracket. Gene Ontology (GO) term enrichment analyses were performed for each main cluster with all terms belonging to the category ‘molecular function’. GO terms showing an adjusted P value ≤ 0.05 were considered significantly enriched compared to all detected transcripts (hypergeometric test followed by Benjamini and Hockberg multiple testing correction). Colour scale (white to orange) indicates enrichment significance. Few non-significant GO terms are displayed for clarity.

When grouping populations by area, a total of 416 transcripts were found significantly differentially transcribed in any area compared to the susceptible reference strain (FC > 3 and adjusted P value < 0.001). Differential expression was mainly found in the agriculture area where 375 transcripts were found differentially expressed while low pesticide and urban areas showed less variation (65 and 5 transcripts respectively). Within each area, transcription ratios were well balanced between over- and under-transcribed genes with most variations being within 20 fold. Clustering analysis based on expression profile across the three areas confirmed the strong variations observed in the agriculture area compared to other environments (Figure 2 and Additional file 3). Gene ontology term enrichment analysis performed on each main cluster revealed an enrichment of multiple GO terms. Cluster 2 representing genes over-transcribed in agriculture areas was enriched in GO terms linked to receptors, oxido-reductases including cytochrome P450 monooxygenases and transcription factors. Cluster 5, representing genes specifically under-transcribed in agriculture area was enriched in the GO term ‘nucleic acid binding’ linked to transcription factors. Clusters 6 to 9 representing genes under-transcribed in all areas were enriched in genes and GO terms related to sugar and protein catabolism, energy storage, and odorant binding proteins (‘odorant binding’).

**Figure 3:**
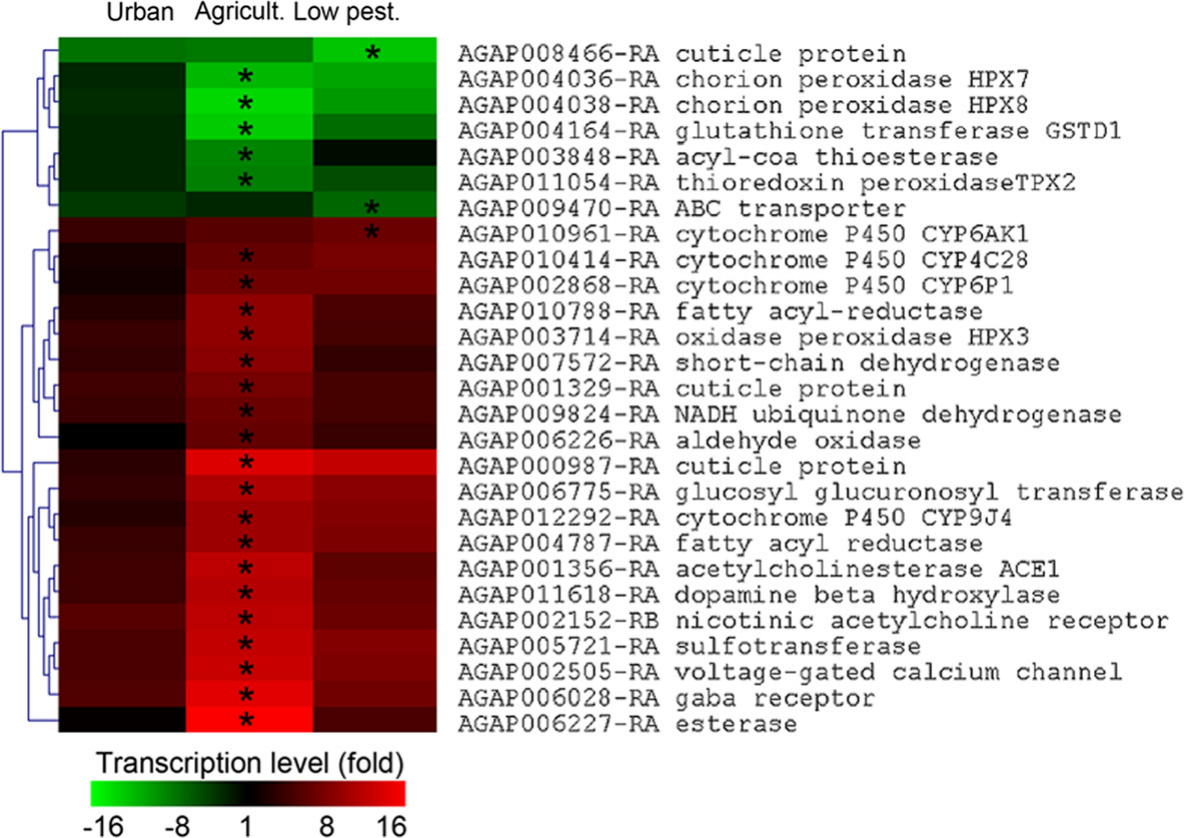
**Candidate genes differentially transcribed in any area.** Clustering analysis was performed on the 27 transcripts showing a significant differential expression in any area and potentially involved in insecticide resistance. Clustering of transcripts was based on differences between mean Log_2_ ratios in each area versus reference using Euclidean distance and complete linkage algorithm. Colour scale (green to red) indicates fold change versus reference strain. Stars indicate a significant differential transcription compared to the susceptible reference strain Ifakara (P ≤ 0.001 and FC ≥ 3).

Focusing on transcripts encoding proteins putatively involved in insecticide resistance (*i.e*. detoxification enzymes *sensu lato*, ABC transporters, insecticide targets, cuticle proteins) confirmed the strong response of populations from agriculture area with 19 transcripts being significantly over-transcribed compared to the susceptible strain (Figure 3). These include three P450s (CYP6P1, CYP9J4, CYP4C28), two cuticle proteins (AAGP001329 and AGAP000987), one esterase (AGAP006227), one UDPGT (AGAP006775), one sulfotransferase (AGAP005721) and one aldehyde oxidase (AGAP006775). The GABA and nicotinic acetylcholine receptors were also over-transcribed in mosquitoes from agriculture areas.

**Figure 4:**
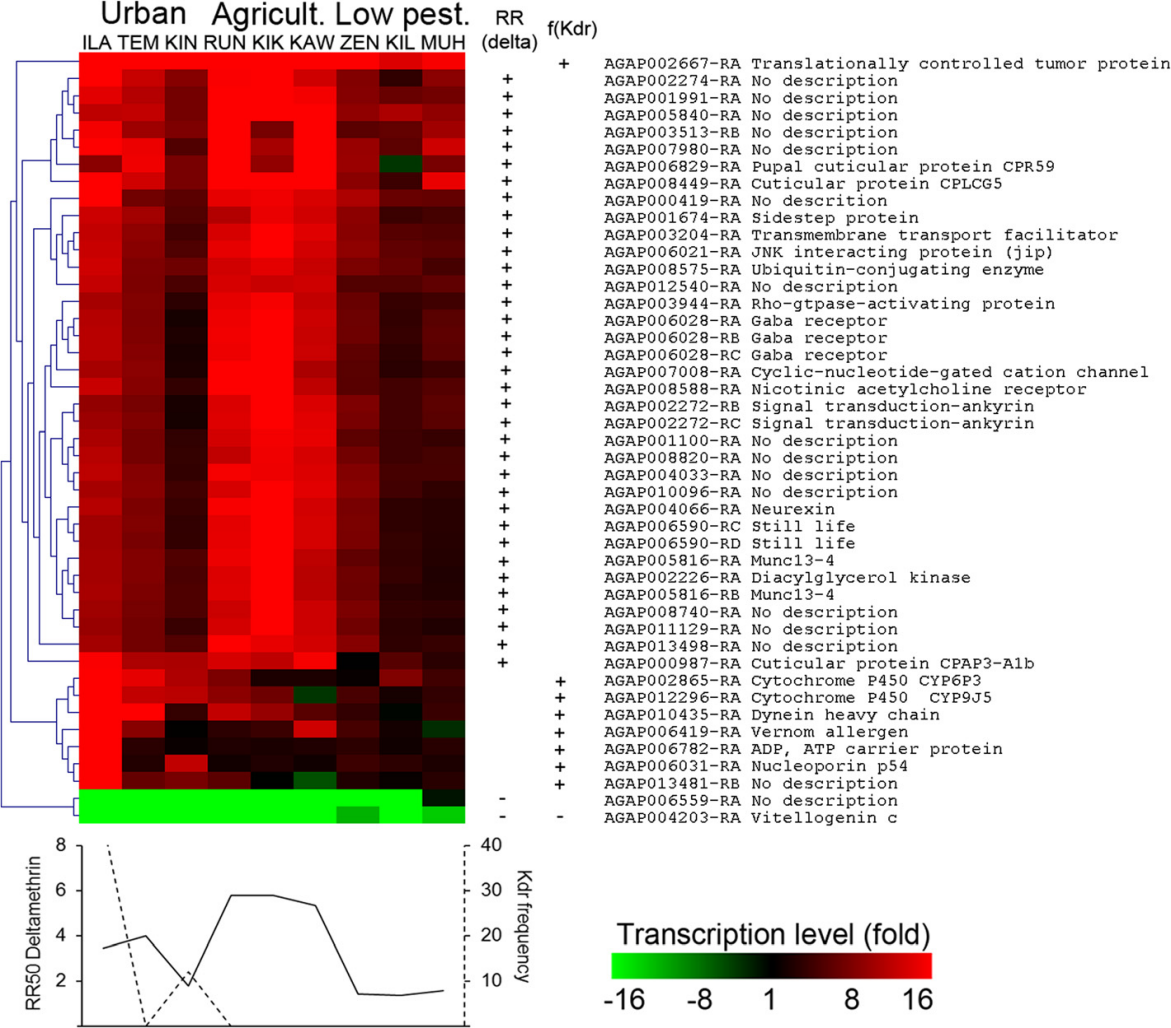
**Genes with expression profiles associated with deltamethrin resistance.** Clustering analysis was performed on the 46 transcripts showing a correlation between their expression profile and deltamethrin resistance (RR_50_) and kdr mutation frequency. Clustering of transcripts was based on differences between mean Log_2_ ratios in each population versus reference using Euclidean distance and complete linkage algorithm. Colour scale (green to red) indicates fold change versus reference strain. + and - indicate a positive (r > 0.85) or negative (r < -0.85) correlation with deltamethrin resistance or kdr mutation frequency.

At the population level, looking for genes whose expression profiles were positively or negatively correlated to insecticide resistance levels in adults identified multiple transcripts correlated to pyrethroid selection pressure (Figure 4). Several genes were correlated to deltamethrin resistance including the GABA receptor (AGAP006028) and the nicotinic acetylcholine receptor (AGAP008588), the ‘still life’ gene (AGAP006590) and two genes encoding cuticle proteins (AGAP006829 and AGAP008449). Although *kdr* mutations were absent in *An. arabiensis* specimens used for microarray study, correlation between gene expression profiles and *kdr* mutation frequencies recorded at the population level were investigated based on the assumption that such association may still identify genes associated with pyrethroid selection pressure. Indeed, height transcripts were correlated to *kdr* mutation frequency obtained from whole populations, including two known P450 candidates (CYP6P3 and CYP9J5), the cuticle gene AGAP000987 and the gene AGAP002667 coding for a protein homologous to a tumour-related protein. Finally, only two genes had their expression profile negatively correlated with deltamethrin resistance or *kdr* mutation, including the transcript AGAP004203 encoding for vitellogenin C. Cross-validation of expression profiles of the cuticle protein coding gene AGAP000987 and the cytochrome P450 CYP6P3 revealed a good correlation between microarray and RT-qPCR although microarray transcription ratios appear slightly over-estimated (Additional file 4). These data confirmed the marked over-transcription of these two genes in urban and agriculture areas compared to the reference strain.

## Discussion

The present study aimed at investigating insecticide resistance levels and identifying associated mechanisms across multiple *An. gambiae* populations from different environments. The eastern Tanzania region appeared suitable such study since insecticide resistance levels are currently increasing in this region with a significant heterogeneity observed in populations from contrasted environments. Although the experimental design (nine populations across three areas of contrasted environments) was constrained by field limitations, results support the role of environment in shaping the distribution and mechanisms of insecticide resistance of malaria vectors in Tanzania.

### Species distribution and insecticide resistance

Most sampled mosquitoes were identified as *An. arabiensis* with *An. gambiae s.s*. only present in some urban populations of Dar es Salaam. Even though a decline of the traditional malaria vector *An. gambiae s.s*. is observed in Tanzania, residual malaria transmission remains due to its replacement by the more exophagic *An. arabiensis* throughout the country [48]. The present study confirmed the preference of *An. gambiae s.s*. for permanent urban breeding sites [49], such as the Jangwani Valley in Dar es salaam and its potency to breed in organically polluted sites while *An. arabiensis* was mainly represented in temporary rural breeding sites [50] including those from intensive agriculture areas [17].

Overall, moderate DDT resistance was found in larvae and adults in populations from the urban area of Dar Es Salaam while deltamethrin resistance was highest in the agriculture area of the Kilimanjaro region. Deltamethrin resistance ratios were higher in larvae than adults although resistance profile across areas was conserved between life stages. The link between agriculture and pyrethroid resistance was confirmed by a significant correlation between deltamethrin resistance levels and agriculture intensity across all populations. Urban populations containing *An. gambiae s.s*. individuals carrying the *L1014S kdr* showed the highest resistance levels to DDT. Indeed, several studies confirmed the strong genetic association between DDT resistance and this *kdr* mutation [15,22,51]. In contrast, *An. arabiensis* populations found in the agriculture area showed higher resistance levels to deltamethrin together with an absence of *kdr* mutation frequencies, suggesting that other resistance mechanisms are predominant. Although all populations showed 100% mortality after 1 h exposure of adults to bendiocarb, 10 min exposure mortality data and larval bioassays suggest that some individuals from urban and agriculture areas may carry resistance alleles. This was expected as carbamates are commonly used in agriculture and are also available for domestic use as revealed by a survey through local dealers (Theresia Nkya unpublished). In addition, it is likely that some metabolic resistance mechanisms selected by insecticides used for vector control are also conferring resistance to carbamates [52]. Such findings highlight the need for investigating background resistance and cross-resistance mechanisms in mosquito populations before switching to carbamate insecticides in high resistance areas.

### Transcription variations associated to different environments

A 15 k *An. gambiae* microarray was used to investigate transcription level variations associated to different environments and insecticide resistance across sampled populations. Because of the high prevalence of *An. arabiensis* in our samples and potential gene expression variations between the two sibling species, only individuals identified as *arabiensis* were used for comparison with the susceptible *An. arabiensis* strain Ifakara. In order to focus on inherited variations and minimize transient variations linked to different environments or sampling bias, bioassays and gene expression studies were performed on F_1_ individuals (*i.e*. progeny of those collected from the field) bred in standard laboratory conditions.

Most transcription level variations compared to the susceptible strain were found in populations from the agriculture area suggesting that the intense use of agrochemicals represent a significant selection pressure for mosquito populations. Such hypothesis was confirmed by an enrichment of GO terms related to detoxification and receptors in genes over-transcribed in this area. Of particular interest is the strong over transcription of three known insecticide targets: the acetylcholinesterase (targeted by organophosphates and carbamates), the GABA receptor (targeted by cyclodienes) and the nicotinic acetylcholine receptor (targeted by neonicotinoids). Increased gene copy number of insecticide receptors has frequently been associated to resistance and often lead to their over-expression in resistant populations [53-57]. The fact that organophosphates and cyclodienes are only used for crop protection in this area supports previous studies highlighting the potential of agricultural pesticides to select for resistance in mosquitoes [18]. Multiple detoxification enzymes were over-transcribed in the agriculture area including the alpha esterase AGAP006227, the UDPGT AGAP006775 and the cytochrome P450s CYP9J4 and CYP6P1. The *Aedes aegypti* paralog of this esterase (AAEL010389) has been found over-transcribed in pyrethroid resistant strains [58] and in response to xenobiotic exposure [34]. Similarly, two *Ae. aegypti* paralogs of this UDPGT (AAEL008560 and AAEL014371) were found over-transcribed in insecticide resistant strains [38,59]. Regarding CYP genes, although the role of CYP9Js remains unclear in *Anopheles*, this subfamily has been frequently involved in insecticide resistance in other insects with several of them validated as pyrethroid metabolizers in *Ae. aegypti* and *Drosophila melanogaster*[60-62]. CYP6Ps have also been frequently associated with insecticide resistance with *An. gambiae* CYP6P3 and *An. funestus* CYP6P9 being validated as pyrethroid metabolizers [36,60,63,64]. The over transcription of multiple detoxification enzymes in the agricultural area supports previous findings suggesting that in absence of *kdr* mutation, resistance of *An. gambiae* to pyrethroids in this region mainly relies on metabolic mechanisms favored by agricultural practices [17]. The gene AGAP000987 encoding the cuticle protein CPAP3-A1b was also strongly over-transcribed in the agriculture area. Cuticle modifications are likely involved in the resistance of mosquitoes to insecticides and genes encoding cuticle proteins have been frequently found over-transcribed in pyrethroid resistant populations [18-20]. An over-transcription of this gene has recently been reported in *An. gambiae* populations from West Africa strongly resistant to deltamethrin supporting its role in resistance (Hyacinthe Toe, unpublished data).

Although resistance to DDT and to a lesser extent to deltamethrin occurred in the urban area, very few genes were found differentially transcribed in this area compared to the reference strain. This can be explained by a lower resistance of *An. arabiensis* compared to *An. gambiae s.s*. (carrying the *L1014S kdr* mutation) in this area which were not used for gene expression study. In addition, although several studies revealed that mosquitoes exposed to urban pollutants display an increased tolerance to insecticides through the induction of their detoxification system [18,34,37], such transient effect may not fully express in F_1_ individuals used for the microarray study. Nevertheless, resistance to insecticides in the urban area may not be the sole consequence of the presence of the *kdr* mutation as few detoxification genes, such as the known pyrethroid metabolizer CYP6P3 showed their highest transcription level in urban populations and were correlated to deltamethrin resistance (see below).

Finally, a fraction of candidate genes identified from the agriculture area were also found over-transcribed in the low pesticide area despite a low agriculture activity and moderate usage of insecticides for vector control in this region. Such situation may be the consequence of the migration of resistant alleles to this region due to the relative proximity of the Kilimandjaro agricultural area. Whether these resistant alleles are stable in this area and maintained by the usage of insecticides for vector control or other factors requires further investigations.

### Transcription variations associated to pyrethroid resistance

As bioassay data revealed significant variations in deltamethrin resistance between populations, correlation between gene expression and deltamethrin resistance profiles were investigated across all populations. This analysis identified 46 transcripts showing an expression profile highly correlated to variations of deltamethrin RR_50_ or *kdr* mutation frequency (r > 0.85 or r < -0.85). As expected, most transcripts correlated to deltamethrin resistance or *kdr* mutation frequency were over-transcribed in the agriculture and urban areas respectively. The transcript encoding vitellogenin C protein (AGAP004203) was found negatively correlated to both factors. Vitellogenin is the major yolk protein of most insects including mosquitoes and its negative correlation to deltamethrin resistance may reflect resistance costs linked to energy reallocation and general stress response [65,66]. The transcription profiles of the cytochrome P450s CYP6P3 and CYP9J5 were positively correlated to *kdr* mutation frequency supporting their role in deltamethrin resistance in Tanzania. Indeed, CYP6P3 has been showed to metabolize both permethrin and deltamethrin [67]. *Anopheles gambiae* CYP9J5 has been previously found over-transcribed in pyrethroid resistant populations [35,68] and various *Ae. aegypti* CYP9Js were shown to metabolize pyrethroids [61,62]. Unexpectedly, CYP6M2 often presented as the *Anopheles* P450 mainly responsible for metabolic resistance to pyrethroids and also metabolizing DDT [44,60,69] was not found significantly upregulated in any area compared to the susceptible reference strain nor correlated to deltamethrin resistance or *kdr* mutation frequency. This supports the importance of other detoxification enzymes in pyrethroid resistance in East Africa. Multiple genes encoding proteins involved in synapse functioning also had their transcription profiles positively correlated to deltamethrin resistance. This includes the *Still life* gene (AGAP006590) encoding a protein that converts GTPase from inactive to active form and modulates synapse differentiation in *Drosophila*[70] and the other GTPase activating protein AGAP00394. Genes encoding the protein *Sidestep* (AGAP001674), the neurexin AGAP004066, the JNK interacting protein AGAP006021, the cyclic-nucleotide-gated calcium channel AGAP007008, the *Munc*-*13* protein AGAP005816, the nicotinic acetylcholine receptor AGAP008588 and the GABA receptor AGAP006028, all linked to synapse functioning and neurotransmitter activity were also found correlated to deltamethrin resistance [71-73]. Taken together, these results suggest that elevated neurotransmitter activity can contribute to insecticide resistance in mosquitoes as previously suggested [74]. Finally, three genes encoding cuticle proteins had their expression profiles correlated to deltamethrin RR_50_ variations (CPR59 AGAP006829, CPLCG5 AGAP008449 and CPAP3-A1b AGAP000987), supporting their possible role in pyrethroid resistance through altered insecticide penetration.

## Conclusions

Insecticide resistance is well established in malaria vectors throughout Africa and represents a threat to malaria control. Previously circumscribed to West and Central Africa, resistance has spread all over Africa dramatically. In absence of new insecticides approved for vector control, developing efficient resistance management strategies is critical for the next decade. This study provides ecotoxicological and molecular data supporting the role of the environment in which the vectors are found in shaping insecticide resistance mechanisms. Both agriculture and urban areas are likely favouring the emergence of resistance to insecticides used for vector control as a consequence of different factors. In agriculture areas, the massive usage of pesticides from various chemical families appears to select for metabolic and cuticle resistance mechanisms to a wide range of insecticides including pyrethroids and their potential alternative carbamates. As insecticide classes used for vector control are frequently used earlier for crop protection, this may explain why resistance of malaria vectors to all insecticides develops so rapidly in Africa. In this concern, implementing molecules/formulations showing limited cross-resistance with pesticides used for crop protection is of high interest for resistance management. In urban areas, the high pyrethroid-impregnated nets coverage completed by uncontrolled indoor spraying with insecticides may strongly select for *kdr* mutations and metabolic resistance mechanisms with a potential role of pollutants in favouring the selection of particular detoxification enzymes. Further understanding of how environment modulates the selection and spread of insecticide resistance mechanisms will help reducing vector control failures and improving resistance management strategies.

## Abbreviations

ITN: Insecticide-treated net; IRS: Indoor residual spraying; DDT: 4,4'-(2,2,2trichloroethane-1,1-diyl)bis(chlorobenzene); VGSC: Voltage-gated sodium channel; WHO: World Health Organization; KDT: Knock down time; KD: Knock down; LC: Lethal concentration; RR: Resistance ratio; Kdr: Knock down resistance mutation; gDNA: Genomic DNA; cRNA: complementary amplified RNA; GO: Gene Ontology; RT-qPCR: Reverse transcription and quantitative polymerase chain reaction; P450: cytochrome P450 monooxygenase enzymes; CYP: genes encoding cytochrome P450 monooxygenase; UDPGT: UDP-glycosyltransferase; JNK: c-Jun N-terminal kinase; GABA: Gamma-aminobutyric acid; ILA: Ilala population; TEM: Temeke population; KIN: Kinondoni population; KIK: Kikafu population; RUN: Rundugai population; KAW: Kawaya population; ZEN: Zeneti population; KIL: Kilulu population; MUH: Muheza population.

## Competing interests

The authors disclosed any financial or competing interests.

## Authors’ contributions

T.N. conducted mosquito sampling, bioassays and molecular work, analyzed results and drafted the manuscript. I.A. contributed to sample preparation and data analysis and helped drafting the manuscript. R.P. performed microarray hybridizations and statistical analyses. R.K. and B.M.B. contributed to specie identification and *kdr* mutation detection. S.M. helped drafting the manuscript. W.K. contributed to study design and coordination and helped drafting the manuscript. J.P.D. conceived and coordinated the study, participated in sample preparation and molecular work, analysed results and wrote the manuscript. All authors read and approved the final manuscript.

## Supplementary Material

Additional file 1 Ecological characteristics of sampled population. Click here for file

Additional file 2 **Correlation matrix between environmental factors and insecticide resistance data.** For each pairwise comparison, Pearson correlation (r) coefficient (above diagonal) and its associated adjusted P value ≤ are shown (below diagonal). Two factors were considered significantly correlated if r ≥ 0.8 or ≤ -0.8 and if adjusted P value ≤ 0.05. Source data are shown as a separate sheet. Click here for file

Additional file 3 **Genes significantly differentially transcribed in any area. For each gene, Log**_**2**_** fold changes and adjusted P values are shown.** For each gene, Figure 1 cluster number and belonging to candidates resistance gene list are indicated. Click here for file

Additional file 4 **Cross-validation of microarray data with by RT-qPCR.** Gene transcription data are expressed as fold change versus Ifakara strain. CYP6P3 AGAP002865 data are shown as plain marks. Cuticle protein AGAP000987 data are shown as empty marks. Squares, triangles and circles represent populations from urban, agricultural and low pesticide usage areas respectively. Population names and correlation coefficient between microarray and RT-qPCR fold changes are indicated. Click here for file

## Data deposition

Microarray data have been deposited to ArrayExpress under accession number E-MEXP-3987.

## References

[B1] WHO World Malaria Report 2011 Geneva: World Health Organization http://who.int/malaria/world_malaria_report_2011/wmr2011_summary_keypoints.pdf

[B2] Rugemalila JB Wanga CL Kilama WL Sixth Africa malaria day in 2006: how far have we come after the Abuja Declaration? Malar J 2006 5 102 10.1186/1475-2875-5-102 17090308 PMC1637112

[B3] Mboera LEG Makundi EA Kitua AY Uncertainty in malaria control in Tanzania: Crossroads and challenges for future interventions Am J Trop Med Hyg 2007 77 112 118 18165482

[B4] Govella NJ Chaki PP Geissbuhler Y Kannady K Okumu F Charlwood JD Anderson RA Killeen GF A new tent trap for sampling exophagic and endophagic members of the *Anopheles gambiae* complex Malar J 2009 8 157 10.1186/1475-2875-8-157 19602253 PMC2720981

[B5] Charlwood JD Vij R Billingsley PF Dry season refugia of malaria-transmitting mosquitoes in a dry savannah zone of east Africa Am J Trop Med Hyg 2000 62 726 732 11304064 10.4269/ajtmh.2000.62.726

[B6] Malaria Vector Control and Personal Protection http://whqlibdoc.who.int/trs/WHO_TRS_936_eng.pdf 16623084

[B7] Akogbeto MC Padonou GG Gbenou D Irish S Yadouleton A Bendiocarb, a potential alternative against pyrethroid resistant *Anopheles gambiae* in Benin West Africa. Malar J 2010 9 204 10.1186/1475-2875-9-204 PMC2912925 20630056

[B8] Sharp BL Kleinschmidt I Streat E Maharaj R Barnes KI Durrheim DN Ridl FC Morris N Seocharan I Kunene S La Grange SS Mthembu JD Maartens F Mertin CL Barreto A Seven years of regional malaria control collaboration - Mozambique, South Africa, and Swaziland Am J Trop Med Hyg 2007 76 42 47 17255227 PMC3749812

[B9] Protopopoff N Matowo J Malima R Kavishe R Kaaya R Wright A West PA Kleinschmidt I Kisinza W Mosha FW Rowland M High level of resistance in the mosquito *Anopheles gambiae* to pyrethroid insecticides and reduced susceptibility to bendiocarb in north-western Tanzania Malar J 2013 12 149 10.1186/1475-2875-12-149 23638757 PMC3655935

[B10] Davies TGE Field LM Usherwood PNR Williamson MS DDT, pyrethrins, pyrethroids and insect sodium channels IUBMB Life 2007 59 151 162 10.1080/15216540701352042 17487686

[B11] Fukuto TR Mechanism of action of organophosphorus and carbamate insecticides Environ Health Perspect 1990 87 245 254 2176588 10.1289/ehp.9087245 PMC1567830

[B12] Ranson H Abdallah H Badolo A Guelbeogo WM Kerah-Hinzoumbe C Yangalbe-Kalnone E Sagnon NF Simard F Coetzee M Insecticide resistance in *Anopheles gambiae*: data from the first year of a multi-country study highlight the extent of the problem Malar J 2009 8 299 10.1186/1475-2875-8-299 20015411 PMC2804687

[B13] Casimiro S Coleman M Hemingway J Sharp B Insecticide resistance in *Anopheles arabiensis* and *Anopheles gambiae* from Mozambique J Med Entomol 2006 43 276 282 10.1603/0022-2585(2006)043[0276:IRIAAA]2.0.CO;2 16619611

[B14] Hargreaves K Koekemoer LL Brooke BD Hunt RH Mthembu J Coetzee M *Anopheles funestus* resistant to pyrethroid insecticides in South Africa Med Vet Entomol 2000 14 181 189 10.1046/j.1365-2915.2000.00234.x 10872862

[B15] Protopopoff N Verhaeghen K Van Bortel W Roelants P Marcotty T Baza D D'Alessandro U Coosemans M A significant increase in *kdr* in *Anopheles gambiae* is associated with an intensive vector control intervention in Burundi highlands Trop Med Int Health 2008 13 1479 1487 10.1111/j.1365-3156.2008.02164.x 18983277

[B16] Kabula B Tungu P Matowo J Kitau J Mweya C Emidi B Masue D Sindato C Malima R Minja J Msanqi S Njau R Mosha F Magesa S Kisinza W Susceptibility status of malaria vectors to insecticides commonly used for malaria control in Tanzania Trop Med Int Health 2012 17 742 750 10.1111/j.1365-3156.2012.02986.x 22519840

[B17] Matowo J Kulkarni MA Mosha FW Oxborough RM Kitau JA Tenu F Rowland M Biochemical basis of permethrin resistance in *Anopheles arabiensis* from Lower Moshi, north-eastern Tanzania Malar J 2010 9 193 10.1186/1475-2875-9-193 20609220 PMC3224900

[B18] Nkya TE Akhouayri I Kisinza W David JP Impact of environment on mosquito response to pyrethroid insecticides: facts, evidences and prospects Insect Biochem Mol Biol 2013 43 407 416 10.1016/j.ibmb.2012.10.006 23123179

[B19] Wood OR Hanrahan S Coetzee M Koekemoer LL Brooke DB Cuticle thickening associated with pyrethroid resistance in the major malaria vector *Anopheles funestus* Parasit Vector 2010 3 67 10.1186/1756-3305-3-67 PMC2924294 20684757

[B20] Chengyuan P Yun Z Jianchu M The clone of laccase gene and its potential function in cuticular penetration resistance of *Culex pipiens pallens* to fenvalerate Pestic Biochem Physiol 2009 93 105 111 10.1016/j.pestbp.2008.12.003

[B21] Martinez-Torres D Chandre F Williamson MS Darriet F Berge JB Devonshire AL Guillet P Pasteur N Pauron D Molecular characterization of pyrethroid knockdown resistance (kdr) in the major malaria vector *Anopheles gambiae s.s* Insect Mol Biol 1998 7 179 184 10.1046/j.1365-2583.1998.72062.x 9535162

[B22] Ranson H Jensen B Vulule JM Wang X Hemingway J Collins FH Identification of a point mutation in the voltage-gated sodium channel gene of Kenyan *Anopheles gambiae* associated with resistance to DDT and pyrethroids Insect Mol Biol 2000 9 491 497 10.1046/j.1365-2583.2000.00209.x 11029667

[B23] Brooke BD Kloke G Hunt RH Koekemoer LL Temu EA Taylor ME Small G Hemingway J Coetzee M Bioassay and biochemical analyses of insecticide resistance in southern African *Anopheles funestus* (Diptera: Culicidae) Bull Entomol Res 2001 91 265 272 10.1079/BER2001108 11587622

[B24] Balkew M Ibrahim M Koekemoer LL Brooke BD Engers H Aseffa A Gebre-Michael T Elhassen I Insecticide resistance in *Anopheles arabiensis* (Diptera: Culicidae) from villages in central, northern and south west Ethiopia and detection of *kdr* mutation Parasit Vectors 2010 3 40 10.1186/1756-3305-3-40 20416109 PMC2868498

[B25] Marcombe S Mathieu RB Pocquet N Riaz M-A Poupardin R Selior S Darriet F Reynaud S Yebakima A Corbel V David JP Chandre F Insecticide resistance in the dengue vector *Aedes aegypti* from Martinique: distribution, mechanisms and relations with environmental factors PLoS One 2012 7 e30989 10.1371/journal.pone.0030989 22363529 PMC3283601

[B26] N'Guessan R Corbel V Akogbeto M Rowland M Reduced efficacy of insecticide-treated nets and indoor residual spraying for malaria control in pyrethroid resistance area, Benin Emerg Infect Dis 2007 13 199 206 10.3201/eid1302.060631 17479880 PMC2725864

[B27] Antonio-Nkondjio C Fossog BT Ndo C Djantio BM Togouet SZ Awono-Ambene P Costantini C Wondji CS Ranson H *Anopheles gambiae* distribution and insecticide resistance in the cities of Douala and Yaounde (Cameroon): influence of urban agriculture and pollution Malar J 2011 10 154 10.1186/1475-2875-10-154 21651761 PMC3118161

[B28] Diabate A Baldet T Chandre F Akogbeto M Guiguemde TR Darriet F Brengues C Guillet P Hemingway J Small GJ Hougard JM The role of agricultural use of insecticides in resistance to pyrethroids in *Anopheles gambiae s.l*. in Burkina Faso Am J Trop Med Hyg 2002 67 617 622 12518852 10.4269/ajtmh.2002.67.617

[B29] Antonio-Nkondjio C Atangana J Ndo C Awono-Ambene P Fondjo E Fontenille D Simard F Malaria transmission and rice cultivation in Lagdo, northern Cameroon Trans R Soc Trop Med Hyg 2008 102 352 359 10.1016/j.trstmh.2007.12.010 18295810

[B30] Yadouleton AWM Asidi A Djouaka RF Braima J Agossou CD Akogbeto MC Development of vegetable farming: a cause of the emergence of insecticide resistance in populations of *Anopheles gambiae* in urban areas of Benin Malar J 2009 8 103 10.1186/1475-2875-8-103 19442297 PMC2686728

[B31] Dinham B Growing vegetables in developing countries for local urban populations and export markets: problems confronting small-scale producers Pest Manag Science 2003 59 575 582 10.1002/ps.654 12741526

[B32] Nwane P Etang J Chouaibou M Toto JC Kerah-Hinzoumbe C Mimpfoundi R Awono-Ambene HP Simard F Trends in DDT and pyrethroid resistance in Anopheles gambiae s.s. populations from urban and agro-industrial settings in southern Cameroon BMC Infect Dis 2009 9 163 10.1186/1471-2334-9-163 19793389 PMC2764715

[B33] Suwanchaichinda C Brattsten LB Effects of exposure to pesticides on carbaryl toxicity and cytochrome P450 activities in *Aedes albopictus* larvae (Diptera : Culicidae) Pest Biochem Physiol 2001 70 63 73 10.1006/pest.2001.2544

[B34] Riaz MA Poupardin R Reynaud S Strode C Ranson H David J-P Impact of glyphosate and benzo a pyrene on the tolerance of mosquito larvae to chemical insecticides. Role of detoxification genes in response to xenobiotics Aquatic Toxicol 2009 93 61 69 10.1016/j.aquatox.2009.03.005 19419775

[B35] Jones CM Toe HK Sanou A Namountougou M Hughes A Diabate A Dabire R Simard F Ranson H Additional selection for insecticide resistance in urban malaria vectors: DDT resistance in *Anopheles arabiensis* from Bobo-Dioulasso Burkina Faso. PLoS One 2012 7 e45995 10.1371/journal.pone.0045995 PMC3457957 23049917

[B36] Djouaka RF Bakare AA Coulibaly ON Akogbeto MC Ranson H Hemingway J Strode C Expression of the cytochrome P450s, CYP6P3 and CYP6M2 are significantly elevated in multiple pyrethroid resistant populations of Anopheles gambiae s.s. from Southern Benin and Nigeria BMC Genomics 2008 9 538 10.1186/1471-2164-9-538 19014539 PMC2588609

[B37] Poupardin R Reynaud S Strode C Ranson H Vontas J David JP Cross-induction of detoxification genes by environmental xenobiotics and insecticides in the mosquito *Aedes aegypti*: Impact on larval tolerance to chemical insecticides Insect Biochem Mol Biol 2008 38 540 551 10.1016/j.ibmb.2008.01.004 18405832

[B38] Poupardin R Riaz MA Jones CM Chandor-Proust A Reynaud S David JP Do pollutants affect insecticide-driven gene selection in mosquitoes? Experimental evidence from transcriptomics Aquatic Toxicol 2012 114 49 57 10.1016/j.aquatox.2012.02.001 22406618

[B39] Dongus S Nyika D Kannady K Mtasiwa D Mshinda H Gosoniu L Drescher AW Fillinger U Tanner M Killeen GF Castro MC Urban agriculture and *Anopheles* habitats in Dar es Salaam, Tanzania Geospat Health 2009 3 189 210 19440962 10.4081/gh.2009.220

[B40] Tanzania Commission for AIDS (TACAIDS), Zanzibar AIDS Commission (ZAC), National Bureau of Statistics (NBS), Office of the Chief Government Statistician (OCGS), and ICF International Tanzania HIV/AIDS and Malaria Indicator Survey 2011-12 2013 Dar es Salaam, Tanzania: TACAIDS, ZAC, NBS, OCGS, and ICF International

[B41] Collins FS Drumm ML Cole JL Lockwood WK Vandewoude GF Iannuzzi MC Construction of a general human-chromosome jumping library, with application to cystic-fibrosis Science 1987 235 1046 1049 10.1126/science.2950591 2950591

[B42] Scott JA Brogdon WG Collins FH Identification of single specimens of *Anopheles gambiae* complex by the polymarase chain-reaction Am J Trop Med Hyg 1993 49 520 529 8214283 10.4269/ajtmh.1993.49.520

[B43] Bass C Williamson MS Wilding CS Donnelly MJ Field LM Identification of the main malaria vectors in the *Anopheles gambiae* species complex using a TaqMan real-time PCR assay Malar J 2007 6 155 10.1186/1475-2875-6-155 18034887 PMC2213665

[B44] Mitchell SN Stevenson BJ Mueller P Wilding CS Egyir-Yawson A Field SG Hemingway J Paine MJI Ranson H Donnelly MJ Identification and validation of a gene causing cross-resistance between insecticide classes in *Anopheles gambiae* from Ghana Proc Natl Acad Sci USA 2012 109 6147 6152 10.1073/pnas.1203452109 22460795 PMC3341073

[B45] Maere S Heymans K Kuiper M BiNGO: a cytoscape plugin to assess overrepresentation of gene ontology categories in biological networks Bioinformatics 2005 21 3448 3449 10.1093/bioinformatics/bti551 15972284

[B46] Poupardin R Riaz MA Vontas J David JP Reynaud S Transcription profiling of eleven cytochrome P450s potentially involved in xenobiotic metabolism in the mosquito *Aedes aegypti* Insect Mol Biol 2010 19 185 193 10.1111/j.1365-2583.2009.00967.x 20041961

[B47] Pfaffl MW A new mathematical model for relative quantification in real-time RT-PCR Nucleic Acids Res 2001 29 e45 10.1093/nar/29.9.e45 11328886 PMC55695

[B48] Russell TL Govella NJ Azizi S Drakeley CJ Kachur SP Killeen GF Increased proportions of outdoor feeding among residual malaria vector populations following increased use of insecticide-treated nets in rural Tanzania Malar J 2011 10 80 10.1186/1475-2875-10-80 21477321 PMC3084176

[B49] Awolola TS Oduola AO Obansa JB Chukwurar NJ Unyimadu JP *Anopheles gambiae* s.s. breeding in polluted water bodies in urban Lagos, southwestern Nigeria J Vector Borne Dis 2007 44 241 244 18092529

[B50] Fournet F Cussac M Ouari A Meyer P-E Toe HK Gouagna L-C Dabire RK Diversity in anopheline larval habitats and adult composition during the dry and wet seasons in Ouagadougou (Burkina Faso) Malar J 2010 9 78 10.1186/1475-2875-9-78 20298619 PMC2907872

[B51] Ramphul U Boase T Bass C Okedi LM Donnelly MJ Mueller P Insecticide resistance and its association with target-site mutations in natural populations of *Anopheles gambiae* from eastern Uganda Trans R Soc Trop Med Hyg 2009 103 1121 1126 10.1016/j.trstmh.2009.02.014 19303125

[B52] Zhao GY Rose RL Hodgson E Roe RM Biochemical mechanisms and diagnostic microassays for pyrethroid, carbamate, and organophosphate insecticide resistance/cross-resistance in the tobacco budworm, *Heliothis virescens* Pest Biochem Physiol 1996 56 183 195 10.1006/pest.1996.0072

[B53] Lee SH Kwon DH Friedberg F Gene Duplication in Insecticide Resistance, Gene Duplication 2011 InTech, Available from: http://cdn.intechweb.org/pdfs/21913.pdf ISBN: 978-953-307-387-

[B54] Bass C Field LM Gene amplification and insecticide resistance Pest Manag Science 2011 67 886 890 10.1002/ps.2189 21538802

[B55] Bariami V Jones CM Poupardin R Vontas J Ranson H Gene amplification, ABC transporters and cytochrome P450s: unraveling the molecular basis of pyrethroid resistance in the dengue vector, Aedes aegypti PLoS Negl Trop Dis 2012 6 e1692 10.1371/journal.pntd.0001692 22720108 PMC3373657

[B56] Wondji CS Irving H Morgan J Lobo NF Collins FH Hunt RH Coetzee M Hemingway J Ranson H Two duplicated P450 genes are associated with pyrethroid resistance in *Anopheles funestus*, a major malaria vector Genome Res 2009 19 452 459 19196725 10.1101/gr.087916.108 PMC2661802

[B57] Djogbenou L Labbe P Chandre F Pasteur N Weill M Ace-I duplication in *Anopheles gambiae*: a challenge for malaria control Malar J 2009 8 70 10.1186/1475-2875-8-70 19374767 PMC2679766

[B58] Strode C Wondji CS David JP Hawkes NJ Lumjuan N Nelson DR Drane DR Karunaratne SHPP Hemingway J Black WC Ranson H Genomic analysis of detoxification genes in the mosquito *Aedes aegypti* Insect Biochem Mol Biol 2008 38 113 123 10.1016/j.ibmb.2007.09.007 18070670

[B59] Riaz MA Chandor-Proust A Dauphin-Villemant C Poupardin R Jones CM Régent-Kloeckner M David JP Reynaud S Molecular mechanisms associated with increased tolerance to the neonicotinoid insecticide imidacloprid in the dengue vector *Aedes aegypti* Aquatic Toxicol 2013 126 326 337 10.1016/j.aquatox.2012.09.010 23058251

[B60] David JP Ismail HM Chandor-Proust A Paine MJI Role of cytochrome P450s in insecticide resistance: impact on the control of mosquito-borne diseases and use of insecticides on Earth Phil Trans Royal Soc B 2013 368 20120429 10.1098/rstb.2012.0429 PMC3538419 23297352

[B61] Pavlidis P Jensen JD Stephan W Stamatakis A A critical assessment of storytelling: gene ontology categories and the importance of validating genomic scans Mol Biol Evol 2012 29 3237 3248 10.1093/molbev/mss136 22617950

[B62] Stevenson BJ Pignatelli P Nikou D Paine MJI Pinpointing P450s associated with pyrethroid metabolism in the dengue vector, Aedes aegypti: developing new tools to combat insecticide resistance PLoS Negl Trop Dis 2012 6 e1585 10.1371/journal.pntd.0001585 22479665 PMC3313934

[B63] Mueller P Chouaibou M Pignatelli P Etang J Walker ED Donnelly MJ Simard F Ranson H Pyrethroid tolerance is associated with elevated expression of antioxidants and agricultural practice in *Anopheles arabiensis* sampled from an area of cotton fields in Northern Cameroon Mol Ecol 2008 17 1145 1155 18179425 10.1111/j.1365-294X.2007.03617.x

[B64] Amenya DA Naguran R Lo TCM Ranson H Spillings BL Wood OR Brooke BD Coetzee M Koekemoer LL Over expression of a cytochrome P450 (CYP6P9) in a major African malaria vector, *Anopheles funestus*, resistant to pyrethroids Insect Mol Biol 2008 17 19 25 10.1111/j.1365-2583.2008.00776.x 18237281

[B65] Paris M David JP Despres L Fitness costs of resistance to Bti toxins in the dengue vector *Aedes aegypti* Ecotoxicology 2011 20 1184 1194 10.1007/s10646-011-0663-8 21461926

[B66] Selye H Stress and distress Comprehensive therapy 1975 1 9 13 1222562

[B67] Mueller P Warr E Stevenson BJ Pignatelli PM Morgan JC Steven A Yawson AE Mitchell SN Ranson H Hemingway J Paine MIJ Donelly MJ Field-caught permethrin-resistant *Anopheles gambiae* overexpress CYP6P3, a P450 that metabolises pyrethroids PLoS Genet 2008 4 e100286 10.1371/journal.pgen.1000286 PMC2583951 19043575

[B68] Nardini L Christian RN Coetzer N Koekemoer LL DDT and pyrethroid resistance in *Anopheles arabiensis* from South Africa Parasit Vectors 2013 6 229 10.1186/1756-3305-6-229 23924547 PMC3751093

[B69] Stevenson BJ Bibby J Pignatelli P Muangnoicharoen S O'Neill PM Lian L-Y Mueller P Nikou D Steven A Hemingway J Sutcliffe MJ Paine MJ Cytochrome P450 6 M2 from the malaria vector *Anopheles gambiae* metabolizes pyrethroids: Sequential metabolism of deltamethrin revealed Insect Biochem Mol Biol 2011 41 492 502 10.1016/j.ibmb.2011.02.003 21324359

[B70] Sone M Hoshino M Suzuki E Kuroda S Kaibuchi K Nakagoshi H Saigo K Nabeshima Y Hama C Still life, a protein in synaptic terminals of Drosophila homologous to GDP-GTP exchangers Science 1997 275 543 547 10.1126/science.275.5299.543 8999801

[B71] Whitmarsh AJ The JIP family of MAPK scaffold proteins Biochem Soc Trans 2006 34 828 832 17052208 10.1042/BST0340828

[B72] Chen EY Marre O Fisher C Schwartz G Levy J Da Silviera RA Berry MJ II Alert response to motion onset in the retina J Neurosc 2013 33 120 132 10.1523/JNEUROSCI.3749-12.2013 PMC3711149 23283327

[B73] Biswas S Russell RJ Jackson CJ Vidovic M Ganeshina O Oakeshott JG Claudianos C Bridging the synaptic gap: Neuroligins and Neurexin I in *Apis mellifera* PLoS One 2008 3 e3542 10.1371/journal.pone.0003542 18974885 PMC2570956

[B74] Kwiatkowska RM Platt N Poupardin R Irving H Dabire RK Mitchell S Jones CM Diabate A Ranson H Wondji CS Dissecting the mechanisms responsible for the multiple insecticide resistance phenotype in *Anopheles gambiae* s.s., M form, from Vallee du Kou, Burkina Faso Gene 2013 519 98 106 10.1016/j.gene.2013.01.036 23380570 PMC3611593

