# Supplementary material for: Insecticide resistance mechanisms associated with different environments in the malaria vector Anopheles gambiae: a case study in Tanzania
Source: Malar J. 2014 Jan 25;13:28. doi: 10.1186/1475-2875-13-28 (PMC3913622; doi:10.1186/1475-2875-13-28)
Supplement: Additional file 1 — Ecological characteristics of sampled population. [file 1475-2875-13-28-S1.doc]

**Additional file 1**. Ecological characteristics of sampled population

| **Area** | **Pop** | **GPS**  **coordinates** | **BS type** | **BS surroundings** | **ITN coverage** | **Use of pesticides in agriculture** | **Urbanization** | **Malaria prevalence** | **Vegetation coverage** |
| --- | --- | --- | --- | --- | --- | --- | --- | --- | --- |
| Urban | ILA | S6.84643 E39.18285 | Valley | Industrial | High | Low | High | Medium | Low |
|  | TEM | S6.87877 E39.24557 | Small scale farms | Small farm | High | Medium | High | Medium | Very low |
|  | KIN | S6.71377 E39.21003 | Drainage systems | Busy road | High | Low | High | Medium | Low |
| Non-polluted | ZEN | S5.21931 E38.65845 | Temporary pools | Residential | Medium | Very low | Very low | Low | Medium |
|  | KIL | S5. 13266 E38.39519 | Containers | Farm | Medium | Very low | Very low | Low | Very low |
|  | MUH | S 5. 10242 E38.4755 | Swamp | Residential | High | Very low | Low | Low | Medium |
| Agriculture | RUN | S3.35043 E37.15805 | Rice field | Farm | Very low | heavy | Very low | Very Low | Medium |
|  | KIF | S3.43737 E37.29678 | Irrigation channels | Rice field | Medium | heavy | Low | Very Low | High |
|  | KAW | S3.43086 E37.27103 | Cowsheds  /cow prints | Rice field | Very low | heavy | Low | Very Low | Absent |

BS: breeding site, ITN: insecticide treated nets
