# Supplementary figures and images for: Insecticide resistance mechanisms associated with different environments in the malaria vector Anopheles gambiae: a case study in Tanzania
Source: Malar J. 2014 Jan 25;13:28. doi: 10.1186/1475-2875-13-28 (PMC3913622; doi:10.1186/1475-2875-13-28)

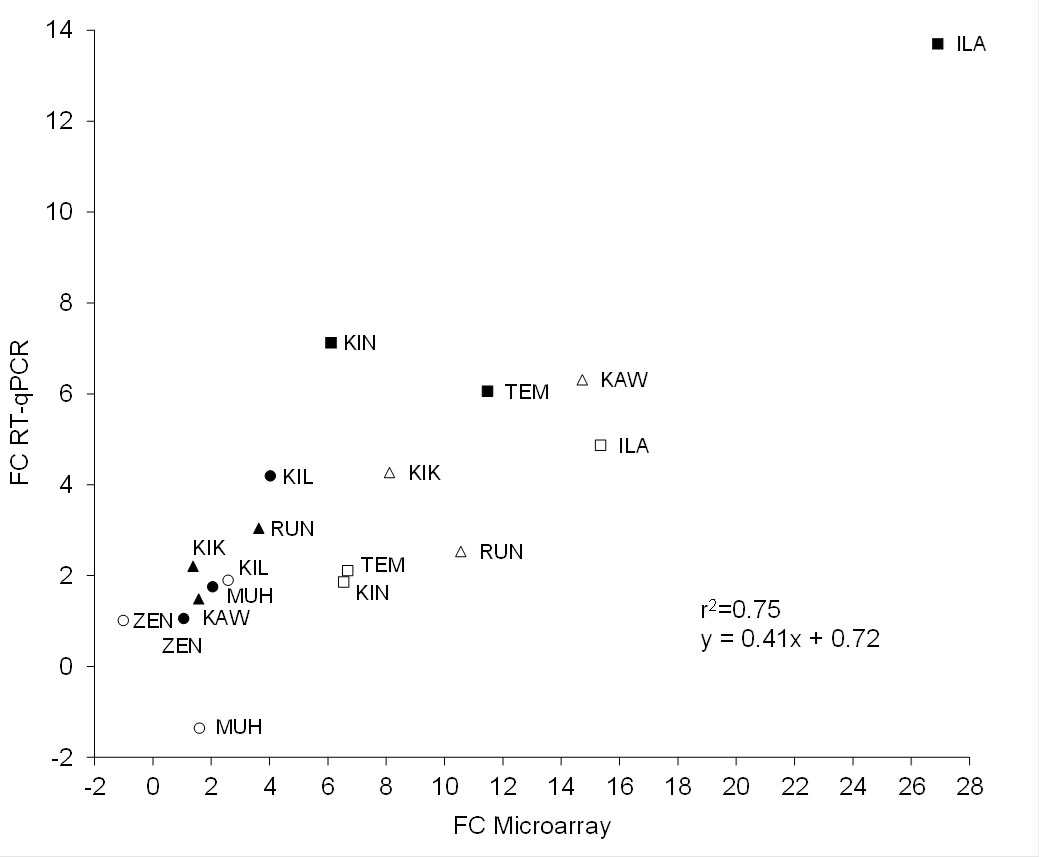

Supplement: Additional file 4 — Cross-validation of microarray data with by RT-qPCR. Gene transcription data are expressed as fold change versus Ifakara strain. CYP6P3 AGAP002865 data are shown as plain marks. Cuticle protein AGAP000987 data are shown as empty marks. Squares, triangles and circles represent populations from urban, agricultural and low pesticide usage areas respectively. Population names and correlation coefficient between microarray and RT-qPCR fold changes are indicated. [file 1475-2875-13-28-S4.tiff]
